# Detection and characterization of bioaerosol emissions from wastewater treatment plants: Challenges and opportunities

**DOI:** 10.3389/fmicb.2022.958514

**Published:** 2022-11-10

**Authors:** Jianghan Tian, Cheng Yan, Sonia Garcia Alcega, Francis Hassard, Sean Tyrrel, Frederic Coulon, Zaheer Ahmad Nasir

**Affiliations:** ^1^School of Chemistry, University of Bristol, Bristol, United Kingdom; ^2^School of Water, Energy and Environment, Cranfield University, Cranfield, United Kingdom; ^3^School of Environmental Studies, China University of Geosciences, Wuhan, China; ^4^School of Physical Sciences, The Open University, Walton Hall, Milton Keynes, United Kingdom; ^5^Institute for Nanotechnology and Water Sustainability, University of South Africa, Johannesburg, South Africa

**Keywords:** bioaerosol, wastewater treatment plant, real time detection, emission characteristics, *in situ* measurement

## Abstract

Rapid population growth and urbanization process have led to increasing demand for wastewater treatment capacity resulting in a non-negligible increase of wastewater treatment plants (WWTPs) in several cities around the world. Bioaerosol emissions from WWTPs may pose adverse health risks to the sewage workers and nearby residents, which raises increasing public health concerns. However, there are still significant knowledge gaps on the interplay between process-based bioaerosol characteristics and exposures and the quantification of health risk which limit our ability to design effective risk assessment and management strategies. This review provides a critical overview of the existing knowledge of bioaerosol emissions from WWTPs including their nature, magnitude and size distribution, and highlights the shortcoming associated with existing sampling and analysis methods. The recent advancements made for rapid detection of bioaerosols are then discussed, especially the emerging real time detection methods to highlight the directions for future research needs to advance the knowledge on bioaerosol emissions from WWTPs.

## Introduction

Rapid population growth and urbanization increased industrial process and other economic activities are all contributing to significant increase of wastewater generation, which is associated with a growing number of wastewater treatment plants (WWTPs) in cities. These WWTPs are often in close proximity to residential areas (Mateo-Sagasta et al., 2015; IWA and OFID, 2018; OECD, 2019). Wastewater treatment generally undergoes pre-treatment, secondary treatment and advance treatment, while sludge is normally treated by dewatering process followed by stabilization prior to being used or disposed of in a WWTP (Korzeniewska, 2011; Mateo-Sagasta et al., 2015). Activated sludge and bio-membrane methods within WWTP infrastructures have been widely applied to treat wastewater, which take advantage of the aeration process during treatment.

**Figure 1:**
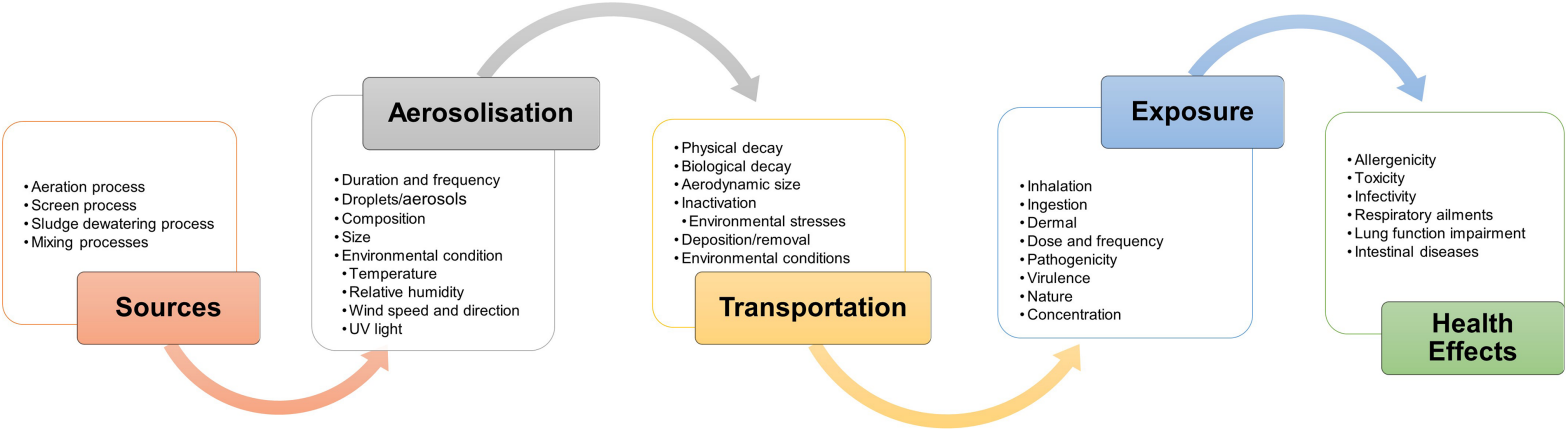
Factors influencing dynamics of bioaerosol and potential health effects from emissions at wastewater treatment plants.

Raw wastewater contains various microorganisms, such as bacteria, virus, fungi, and some of them are pathogenic. During the aeration process, these microorganisms can aerosolize from wastewater to the air forming bioaerosols. These can impact on human health by way of inhalation or ingestion, leading to a range of health effects (allergenicity, toxicity, and infectivity) especially in sewage workers and nearby communities (Gerardi and Zimmerman, 2005; Fracchia et al., 2006; Korzeniewska et al., 2008; Kim et al., 2018) as well directly impacting regional air quality (Upadhyay et al., 2013). Moreover, operations involving mechanical agitation can accelerate the dispersal of bioaerosols (Papke and Ward, 2004). Thus, WWTPs are considered to be an important source of bioaerosol emissions (Michalkiewicz, 2019). Figure 1 illustrates the bioaerosol dynamics from different typical sources at WWTPs and describes how various factors affect aerosolization, transport, dispersal, decay, exposure and potential health effects from emissions at wastewater treatment plants. This information is vital to understanding the processes and factors affecting the WWTPs aerobiology to inform the development and implementation of control measures.

A wide range of microbial species are found within bioaerosol emissions from WWTPs, including heterotrophic/mesophilic bacteria, fungi, and pathogenic organisms (Korzeniewska, 2011), as well as their metabolic products such as endotoxins (1,3)-β-D-glucan molecules and mycotoxins (Garcia-Alcega et al., 2017; Mbareche et al., 2019). Moreover, norovirus and adenovirus are significant viral species among bioaerosol emissions from WWTPs (Masclaux et al., 2014). Furthermore, actinomycetes and fecal coliform bacteria have also been investigated (Vítězová et al., 2012; Li et al., 2013). Since the start of COVID-19 pandemic, there has been growing interest in understanding the fate of SARS-CoV-2 virus in WWTPs and potential transmission through fecal-oral and fecal-inhalation routes (Foladori et al., 2020; Heller et al., 2020), and most importantly, the WWTP associated bioaerosols was considered to hold the potential as the early indicator for future pandemics (Singh et al., 2021).

Air samples collected around mechanical and biological treatment sites in WWTPs have shown microorganisms less than 2 μm in size (Bauer et al., 2002; Kowalski et al., 2017; Hsiao et al., 2020). These fine size fractions of bioaerosols have longer airborne residence time enabling their long-distance transport from their sources and can potentially reach the alveolar region of the respiratory tract. Specifically, pathogenic bacteria, fungi and yeast such as Citrobacter, Enterobacter, Klebsiella, Serratia, Pantoea were identified as being in inhalable range (Filipkowska et al., 2002, 2007, 2008; Korzeniewska et al., 2007, 2008, 2009).

Since the late 1970s, it was reported that several typical symptoms were frequently found on the WWTP’s employees, named ‘sewage worker’s syndrome’ (Rylander et al., 1976; Clark, 1987; Hung et al., 2010; Stellacci et al., 2010). The main characteristics of those symptoms are malaise, fatigue, weakness, headache, dizziness, acute rhinitis, fever, respiratory diseases (Hung et al., 2010; Stellacci et al., 2010). Studies have suggested that there was a strong connection between ‘sewage worker’s syndrome’ and bioaerosol emissions (Cyprowski and Krajewski, 2003; Patentalakis et al., 2008). Nonetheless, further investigation will enhance the evidence base to strengthen the association between casual agents of sewage worker’s exposure and ill health outcomes (Van Hooste et al., 2010; Mbareche et al., 2022). Consequently, concurrent with a growing number of WWTPs, there have been increasing concerns over public and environmental health risks associated with bioaerosol emissions from WWTPs, and the risk assessment of bioaerosols from WWTPs became significant to regulations of workplace safety and community public health (Peccia et al., 2008). However, the evidence base on nature and magnitude of bioaerosol emissions from WWTPs and potential public health impacts remains inconclusive primarily due to methodological constraints associated with diverse sampling and analysis methods, insufficient dose–response data, and varied health endpoints (Tian et al., 2020; Mbareche et al., 2022). Hence, both operators and regulators are facing considerable challenges to devise proportionate risk-based operation and policies to permit efficient management of potential risks to human and environmental health.

This paper aims to review the existing state of knowledge on emission characteristics of bioaerosols from WWTPs, with a view to highlight the shortcoming associated with existing methods, discuss the recent advancements and identify future directions for research to advance the knowledge on bioaerosols emissions from WWTPs.

## State of the art

Overall, the scientific literature on bioaerosol emissions from WWTPs is limited. A review of the literature was conducted to examine the progress on detection and characterization methods of bioaerosols in WWTPs, using major citation databases (Scopus, Web of Science, Google scholar). The studies were grouped according to different sampling methods and analytical approach. Table 1 provides a brief overview of representative studies conducted to understand the nature, magnitude, and size distributions of different analytes during varied sampling duration.

**Table 1:** Emission characteristics of bioaerosol emissions from wastewater treatment plants (WWTPs).

References	Place	Sampling site	Main analytes	Method	Instrument	Sampling duration	Analytical approach	Key findings
Bauer et al. (2002)	Vienna, Austria	Two WWTPs (an activated sludge plant and a fixed-film reactor)	Cultivable bacteria and fungi	Filtration	(Glass manifold) sterile cellulose nitrate filters (Sartorius, 47 mm diameter, 0.45 mm pore size)	About 5 min each	Cultivation	1. Activated sludge plant have a higher concentration of bacterial and fungal aerosols compared to the fixed-film reactor; 2. Majority of particles were smaller than 2.0 μm (respirable range); 3. It was suggested that the surface area of the aeration tank should be decreased.
Brandi et al. (2000)	Northwest Adriatic coast, Italy	Near the wastewater aeration tanks	Bacteria and fungi	Impaction and impingement	The Andersen Six-Stage Viable Particle Sampler, SAS (Surface Air System) impactor and All Glass Impinger	20 min each	Cultivation	1. Mechanical agitation of the sludge generates aerosols with high concentrations of bacteria and fungi; 2. Aerobic digestion with a submerged microbubble system seems to pose little risk from airborne transmission of pathogenic bacteria and fungi to waste-treatment workers and local residents.
Karra and Katsivela (2007)	Crete, Greece	Screens, aerated grit chambers (indoor), primary settling tanks, (partially covered) primary and secondary settling tanks, aeration tanks, chlorination and sludge processors	Mesophilic heterotrophic bacteria (total coliforms, fecal coliforms and Enterococci) and fungi	Impaction	Air sampler MAS 100 (Merck, Germany)	(Did not mention)	Cultivation	1. The highest concentration of bioaerosols were observed at aerated grit chambers (pre-treatment); 2. A gradual decrease of bioaerosol emissions was observed during the advanced wastewater treatment from the pre-treatment to the primary, secondary and tertiary treatment.
Katsivela et al. (2017)	Chania (Crete), Greece	A WWTP (aerated grit removal and primary sedimentation processes)	Bacteria and fungi	Impaction	Andersen six-stage viable particle sampler (Thermo ESM Andersen Instruments GmbH, Germany)	(Did not mention)	Cultivation	1. Proper wastewater treatment is efficient to the reduction of bioaerosol emissions thus decrease the possibility of associated health risks; 2. Findings on significant positive linear relationships between hydrogen sulphide and size-fractionated bacterial aerosol concentrations (and mass concentrations of different particulate matter fractions) may support the possible drift of some heterotrophic bacteria and particulate matter from the wastewater to the atmosphere by the release of the hydrogen sulphide gas.
Kowalski et al. (2017)	Upper Silesia, Poland	Pre-treatment, biological treatment and activated sludge post-processing stages at five different WWTP	Viable airborne bacteria and fungi	Impaction	6-stage Andersen impactor	8 min each for Anderson impactor	Cultivation	1. The highest concentration of bacterial aerosol was found in the activated sludge post-processing and mechanical purifying stages, which was probably caused by the close environment of the sampling (indoor); 2. Dominant size of airborne bacteria: 2.1–3.3 μm (clarifiers and the sludge post-processing); 3.3–4.7 μm (mechanical treatment and aeration tanks).
Li et al. (2013)	Xi’an City, China	Aerated grit chamber, oxidation ditch, secondary settling tank, sludge dewatering house	Airborne viable bacteria, fungi and actinomycetes	Impaction	Andersen six-stage impactor	10 min each for the impactor (three repetitions each time)	Cultivation	1. The area of mechanical agitation of sludge (aeration and sludge thickening operations) is the largest emission source of bacteria and actinomycete aerosols; 2. Majority of bacteria, fungi and actinomycete aerosols were in respirable size range (less than 3.3 μm), which have high potential to cause adverse health effects.
Michalkiewicz (2019)	Wielkopolska Region, Poland	9 WWTPs	Bacteria and fungi	Sedimentation and impaction methods	MAS 100 Eco type air sampler (Merck)	(Did not mention)	Cultivation	1. Coliform bacteria (an important factor to be monitored) reflects the level of air pollution with bioaerosols from sewage; 2. The higher humidity favored the occurrence of a higher abundance of microorganisms.
Niazi et al. (2015)	Tehran, Iran	Four operational units and one background site	Bacteria and fungi	Impaction	QuickTake 30 sample pump and Bio Stage single-stage cascade impactor (SKC, United States)	2 min each, 240 samples in total	Cultivation. Identification: biochemical test for bacteria; microscopic method for fungi	1. Highest air pollution happened in warm season; 2. Significant relationship between environmental parameters and concentrations of bacterial and fungal were observed.
Vítězová et al. (2012)	Czech Republic	I: Near the screen (sand catcher); II: in the middle of activation tank; III: near area for dewatered sludge	Heterotrophic bacteria, fungi, actinomycetes and fecal coliform bacteria	Impaction	MAS-100 (Merck, Germany)	(Did not mention)	Cultivation	The vicinity of sand catcher and activation tanks are at highest risk due to the presence of higher concentration of pathogenic mesophilic bacteria (includes fecal coliform bacteria).
Brisebois et al. (2018)	Eastern Canada	Screening, primary and secondary screening, biofiltration, grit/fats, oils and greases (FOGs) removal unit, and secondary decantation at four WWTPs	Human pathogen viruses	Impaction and filtration	Coriolis^®^μ (Bertin Technologies, Montigny-le-Bretonneux, France), Marple Personal Cascade Impactor (Thermo Fisher Scientific, Waltham, United States) and SASS 2300 sampler (Research International, Washington, United States)	6-h shift for Coriolis^®^μ, 5 h for Marple impactor, 100 min for SASS 2300 sampler	qPCR (molecular method) and viral metagenomic approach	1. Four viral pathogens of particular concern were tested; 2. Human DNA viruses were in much greater relative abundance than viral RNA from bioaerosols from WWTPs.
Carducci et al. (2000)	Livorno, Italy	1. An activated sludge plant; 2. An anaerobic sludge treatment plant; 3. A sewage washing station	Bacteria and virus	Impaction	SAS impactor	(Did not mention)	Cultivation and RT-PCR (molecular method)	1. Highest bioaerosol contamination was found at sewage washing station (high risk); 2. Enteric virus were frequently presented in the bioaerosol emissions, and isolated enteroviruses of human origin represent a significant health hazard, as they can be transmitted by the respiratory route and are known to cause often-serious extra-intestinal pathologies.
Han et al. (2020b)	Beijing, China	A municipal A^2^/O WWTP	Total bacteria, bacteria with *Enterobacteriaceae*, *Staphylococcus aureus*, and *Pseudomonas aeruginosa*.	Impaction	Anderson six sampler	(Did not mention)	Cultivation and MiSeq high-throughput sequencing	1. The bioaerosol generated in the bubble bottom aeration is dominated by <3.3 μm in size, while the brush surface aeration is the opposite; 2. Capping or sealing of major treatment sections can effectively reduce the emission of bioaerosols from WWTP to the surrounding environment.
Han et al. (2019)	Beijing, China	The aerobic tank of a municipal WWTP, which uses an anaerobic/anoxic/oxic process	Submicron aerosols (SA)		A particulate matter sampler (TH-PM1.0–100, TianHong, Wuhan, China)	24 h	MiSeq sequencing, high-throughput sequencing-based metagenomic analysis	1. The unimodal SA size distribution ranges largely from 68 to 350 nm; 2. Antibiotic resistant genes in SA may lead to high risk to human.
Liu et al. (2020)	Shijiazhuang City, China	A municipal WWTP and a pharmaceutical WWTP	Pathogenic and inhalable bacterial aerosol (PM2.5, PM10)	Filtration	Medium-flow continuous filtration air particulate samplers	44 h	Miseq sequencing and PCR technique (molecular/DNA-based approach)	1. Indoor sludge dewatering facilities were the significant sources of bacterial aerosol emissions, and the process was dominated by aerosolization rather than dispersion; 2. Outdoor aerosols have higher similarity to ambient aerosols than wastewater, revealed that dispersion is more important than aerosolization process.
Masclaux et al. (2014)	Zürich, Switzerland	31 WWTPs	Airborne virus (adenovirus, norovirus and the hepatitis E virus)	(Gelatine) filtration	Gelatine filters embedded in standard cassettes (SKC, Inc. Eighty-Four, United States)	At least 1 h	qPCR	1. Adenovirus was present in 100% of summer WWTP samples and 97% of winter samples; 2. Concentrations of potentially pathogenic viral particles in WWTP air are non-negligible and could partly explain the work-related gastrointestinal symptoms often reported in employees in this sector.
Mbareche et al. (2022)	Quebec, Canada	Screening, grit/FOGs removal, settling tank, and biofiltration at eight WWTPs	Endotoxins, total cultivable and gram-negative bacteria and pathogens indicators	Impaction	Six-stage Anderson Impactor (Thermo Fisher Scientific, Waltham, MA, United States), SASS 3100 (Research International, Inc., Monroe, WA, United States)	(Did not mention)	qPCR	1. The screening, grit/FOGs removal and biofiltration were the most bioaerosol-loaded sites; 2. Water temperature is an important factor for microbial activity – increasing the rate of air changes per hour in summer would be beneficial to reduce the concentration of bioaerosols.
Orsini et al. (2002)	Italy	WWTP in a university hospital	Viable bacteria	Impaction	Impacting sampler (surface air system (SAS), PBI)	(Did not mention)	Cultivation, bio-chemical test and AP-PCR (molecular method)	An integrated molecular approach can contribute in studying bacteria air-diffusion and personnel contamination.
Pascual et al. (2003)	Spain	WWTP	Molds and yeasts, total and fecal coliforms	Impaction	Air Sampler MAS 100 impactor (Merck)	(Did not mention)	Cultivation and PCR (molecular method)	1. Heavy bioaerosol pollution was observed at pre-treatment and primary settlers; 2. Wind speed and daily inflow at the WWTP were two important factors.
Ranalli et al. (2000)	Como, Italy	WWTP	Bacteria	Sedimentation and impaction	SAS (Surface Air Systems, PBI) viable sampler, Sartorius MD8	60 min	Cultivation and PCR (molecular method)	The highest bacterial emission rates (total aerobic heterotrophic and fecal coliform bacteria) were registered during the preliminary mechanical treatments.
Thorn et al. (2002)	Sweden	Three WWTP in different cities (sampling sites including pump stations, sludge hall, flocculoreaction room, indoor sedimentation basins etc.)	Airborne bacterial endotoxin	Filtration	Isopore filters (ATTP 0.8um; Millipore, Cambridge, MA). For personal samplers: Gil-Air 3 SC, Gilian personal air sampler, Gilian Instrument Corp., NJ, United States	(Did not mention)	Immunoassay	1. The highest endotoxin concentrations were found around agitation practices of wastewater; 2. The highest endotoxin values were found at worksites located indoors.
Cyprowski et al. (2011)	Poland	A WWTP in a metal industry	(1,3)-β-glucans	Filtration	25-mm-glass-fiber filters GF/F (Whatman, International Ltd., Maidstone, UK) and Button Aerosol Sampler (SKC Inc., Eighty-Four, PA, United States)	(Did not mention)	Immunoassay	Soluble and insoluble (1,3)-β-glucan fractions were suggested to be analyzed in occupational environment.
Hsiao et al. (2020)	Taiwan, China	Aeration area of an urban wastewater treatment plant	Fluorescent particles	*In-situ*	UV-APS	Quadruplicate over 60 min	Fluorescence-based method	1. On a number basis, the full PSD (particle size distribution) exhibits a unimodal distribution and is dominated by nanoparticles (contribute 99% of total concentration); 2. On a volume basis, the distribution exhibits dual peaks, and the mode sizes are 0.5–0.7 μm and 2–3 μm; 3. The volume concentration of submicron particles is 2 times that of supermicron particles; 4. Submicron particles are major contributors to both the number and volume concentration of particles in the WWTP. 5. Most fluorescent particles may be bacterial aggregates or fungal species.
Li et al. (2016)	Beijing, China	7 intra-plant sites within a WWTP	1. Viable bacteria and fungi 2.Fluorescent particles	1. Impaction 2. *In-situ*	1. Reuter Centrifugal Sampler High Flow (RCS) (Biotest, Inc.) 2. UV-APS (ultraviolet aerodynamic particle sizer)	30 min	Fluorescence-based method and PCR	1. The sludge thickening basin and the screen room were the dominant emission sources of bacterial and fungal aerosols; 2. The UV-APS results showed that for most sampling site, the peak of the number size distribution (or number concentration) is in the range of 3–4 μm.
Tian et al. (2020)	Cranfield, UK	A WWTP within a university campus	Fluorescent particles	*In-situ*	Spectral Intensity Bioaerosol Sensor (SIBS)	Around 3–3.5 h (five repeated measurement)	Fluorescence-based method	1. Particle number concentrations were highly variable; 2. The on-site activity affected the particle size distribution and number concentrations; 3. Predominant particles were in fine size scale (less than 1 μm).

### Sampling and analytical methods

To assess the risk of bioaerosols from WWTPs, different sampling and analytical methods have been applied for the qualitative and quantitative studies of bioaerosols. Major sampling methods involve bioaerosols collection through impaction, impingement, filtration, and cyclone followed by a range of post collection analysis methods. Broadly, those methods can be divided into culture-based and culture-independent methods, and the advantages and disadvantages of different sampling and analytical approaches have been intensively discussed in literatures (Mainelis, 2020; Kathiriya et al., 2021). Majority of studies on emission characteristics of bioaerosols were based on cultivation methods (Brandi et al., 2000; Bauer et al., 2002; Karra and Katsivela, 2007; Vítězová et al., 2012; Li et al., 2013; Niazi et al., 2015; Katsivela et al., 2017; Kowalski et al., 2017; Michalkiewicz, 2019). Typical processes of culture-based methods entail three stages including (i) sampling, (ii) incubation and (iii) enumeration. During the sampling stage, viable airborne microorganisms are collected either by impaction, filtration or impingement and transferred onto the culture medium (e.g., agar, depends on the targeted microorganisms; Korzeniewska, 2011; Mainelis, 2020). After sample collection, colonies of bacteria and fungi are incubated on a defined solid media and temperature for a period ranging between 2 and 7 days. The concentration of bioaerosols is then determined by counting colony formed and expressed as colony-forming units (CFUs) per 1 m^3^ (Korzeniewska, 2011; Mainelis, 2020). The culture-based methods for bioaerosols are relatively sensitive and widely used in bioaerosols quantification (Douwes et al., 2003), however, they have some disadvantages such as low repeatability, relying on the incubation conditions and poor time resolution. Further to this and most importantly, there is a vast number of viable but non-culturable (VBNC) microorganisms in the environment, and therefore culture-based methods are leading to a significant underestimation of the actual viable bioaerosol concentrations in air samples. In order to improve quantitative and qualitative analysis of bioaerosols, several culture-independent methods have been developed, such as staining method, immunoassay method, molecular method (e.g., polymerase chain reaction, PCR; Carducci et al., 2000; Ranalli et al., 2000; Orsini et al., 2002; Thorn et al., 2002; Pascual et al., 2003; Cyprowski et al., 2011; Masclaux et al., 2014; Mbareche et al., 2017; Ferguson et al., 2019; Liu et al., 2020). These new methods, specifically that utilize DNA/RNA based approaches along with Next Generation Sequencing (NGS) technologies showed great capability in improving the understanding of identities, distribution, abundance, diversity and function of airborne microbial communities in WWTPs. (Liang et al., 2012; Brisebois et al., 2018; Han et al., 2019; Corpuz et al., 2020; Kabir et al., 2020; Han et al., 2020b; Bhardwaj et al., 2021; Kathiriya et al., 2021; Mbareche et al., 2022). To conclude, the existing evidence base on bioaerosol emission from WWTPs stems from a range of sampling and post-collection analysis methods ranging from culture-based methods to immunoassay and advanced molecular methods. Whilst these have advanced knowledge of bioaerosol emission from WWTPs, harmonization of sampling methods, sampling design and analytic methods is lacking. A decision tree framework will help to evaluate the relevance and utility of different sampling and analytical methods to a specific endpoint.

### Nature and magnitude of bioaerosol emissions

In a study by Niazi et al. (2015), the results showed that *Bacillus*, *Staphylococcus* spp., and *Micrococcus* spp. were the most frequently observed bacteria types in the bioaerosols emitted from WWTPs, while the dominant fungi species were *Cladosporium* spp. and *Penicillium* spp. Similarly, Michalkiewicz et al. (2011) pointed out that *Corynebacterium*, *Bacillus* spp., *Staphylococcus* spp., *Pseudomonas aeruginosa* and *Micrococcus* spp. were the prevalent bacteria in their study and these potentially pathogenic infectious bacteria can pose a serious hazard to onsite workers an nearby communities. Another study done by Breza-Boruta and Paluszak (2007) showed that *Pseudomonas* were the predominant bacteria species. The most occurring species recorded by Kowalski et al. (2017) were Gram-positive cocci and non-sporing Gram-positive rods. Moreover, some by-products of airborne microorganisms’ agents such as (1–3)-β-D glucans and bacterial endotoxin are being measured because of their toxic potency, immunological and allergic reactions through exposure (Thorn et al., 2002; Cyprowski et al., 2011). Additionally, viruses were also investigated as the pathogenic viral particles in WWTP air that can partly explain the work-related symptoms (Masclaux et al., 2014; Brisebois et al., 2018; Corpuz et al., 2020). Additionally, mesophilic heterotrophic bacteria (total coliforms, fecal coliforms, and enterococci), *Escherichia coli* (or *E.coli*) and staphylococci were monitored in the surrounding air at different stages of wastewater treatment since they are the indicators of fecal pollutants in the wastewater (Michalkiewicz, 2019).

Bioaerosol emissions were found to exist in every stage of wastewater treatment, and highly variable during different stages of wastewater treatment (Li et al., 2013; Michalkiewicz, 2019; Han et al., 2020a), with a concentration from 10^2^ to 10^4^ CFU/m^3^ for viable bacterial and fungal aerosols. Moreover, mechanical agitation, aeration tanks and pre-treatment section were generally considered to be the highest concentrations of bioaerosols (Li et al., 2016; Kowalski et al., 2017; Michalkiewicz, 2019). Though many studies have been done to establish the risk assessment procedure at WWTP such as quantitative microbiological risk assessment (QMRA), however, there is no uniform standards for assessing concentrations of airborne microorganisms on the workplace especially for WWTPs. At present, there is only limited regulations about the critical values people were exposed to bioaerosol concentrations. In the United Kingdom Environment Agency (2017), published a guidance about the environmental monitoring strategy of bioaerosols at regulated facilities proposing the use of culture based methods and sample collection by impaction and filtration. Michalkiewicz (2019) pointed out that there are no limit values for microorganisms and endotoxins in the air on worksite in Poland. Overall, the bioaerosol emissions from WWTPs are diverse (including bacteria, fungi, viruses, and secondary metabolites), and concentrations are highly variable depending upon multiple operational and meteorological variables affecting the timing, intensity, spatial extent and duration of emissions.

### Size distribution of bioaerosol emissions

Dominant size ranges of airborne bacteria and fungi ranged between 2.1 and 3.3 μm at clarifiers and sludge post-processing stages, and between 3.3 and 4.7 μm near mechanical treatment and aeration tanks (Kowalski et al., 2017). Li et al. (2013) revealed that majority of bacteria, fungi and actinomycete aerosols were in respirable size range (less than 3.3 μm), which have high potential to cause adverse health effects. Recent work by Hsiao et al. (2020) further indicated that submicron particles are major contributors to both the number and volume concentration of particles in the WWTP.

Bioaerosol particle morphological characteristics such as size distribution, surface area and asymmetry factor (AF, i.e., shape/aspect ratio, or sphere/rod) are also central to understanding emissions and downwind dispersal from source of particles (Vestlund et al., 2014). These morphological characteristics, especially size distribution, highly affect bioaerosol particles behavior and are important factors in predicting their dispersal (Madelin and Johnson, 1992). For example, the deposition rates for bioaerosols and non-biological particles are a function of particle size, rather than the nature of the particle (Kaye et al., 2000; Ho, 2002; Könemann et al., 2019). However, the particle size distributions of bioaerosol emissions are affected by multiple complex mechanisms as the particles will disintegrate into smaller fragments or single spores due to the disturbance/agitation activities of release mechanisms or during the sampling campaign (Madelin and Johnson, 1992), In addition, the emission characteristics of bioaerosols vary with time and process. For instance, the dominant bioaerosol particle size generated in the bubble bottom aeration is dominated by particles less than 3.3 μm in size while the brush surface aeration is particles larger than 3.3 μm (Han et al., 2020b). Whilst the knowledge on size distribution of bioaerosol emissions from different operational stages of WWTPs is limited, the available evidence suggests the dominance of respirable size fraction. This has implications for transport, dispersion, exposure, and resultant health impacts.

### Factors responsible for bioaerosol emissions characteristics

Numerous studies indicated that the concentration of bioaerosols in WWTPs were influenced by the sampling location (Breza-Boruta and Paluszak, 2007), type of wastewater, aeration method, climatic conditions, wastewater treatment equipment, sunlight, wind speed, and relative humidity (Karra and Katsivela, 2007; Michalkiewicz et al., 2011). Specifically, the concentration of fungal aerosols was largely found at pre-treatment, primary treatment and grit chamber stages (Pascual et al., 2003; Kim et al., 2010; Niazi et al., 2015). Michalkiewicz et al. (2011) also reported that several key factors influenced bioaerosols emission from WWTP including: (a) turbulence and tremor in wastewater, (b) wind speed and direction and wind effect level, and (c) rainfall. In contrast to mechanical aeration, the diffusion aeration system undergoes less turbulence (Niazi et al., 2015). There was a significant relationship between environmental parameters and concentrations of bacterial and fungal bioaerosols. Among the different meteorological conditions recorded by Niazi et al. (2015), significant correlations were found between bacteria concentrations and temperature, and fungal concentrations and relative humidity in air. Similarly, Jones and Harrison (2004) reported that temperature and water availability will affect the size and growth of the bioaerosol source material. For instance, the water availability is critical to stimulate the release of fungal spores. Similarly, wind speed can affect the bioaerosol concentration through atmospheric mixing and removal of biological materials from surfaces. In general, a range of wastewater treatment processes along with meteorological and topographical conditions affect the emissions, fate and behavior of bioaerosols.

## Key barriers to advance bioaerosols detection and characterization

As summarized in Table 1, a range of sampling/collection and analysis methods have been applied for the identification and quantification of bioaerosols. Each method has its own advantages and disadvantages. Depending on different analytes and health endpoint, appropriate sampler and analysis approach should be chosen to improve the efficiency of bioaerosols collection for subsequent analysis (Haddrell and Thomas, 2017). However, there are major limitations that impeded our understanding of bioaerosol emissions from WWTPs. For both culture-based and culture-independent methods, the collection efficiency and bio efficiency of different sampling methods are variable and relatively low (Pöhlker et al., 2012). This can largely affect the viability, cultivability, size, and representativeness of sampled particles, which leads to the underestimation of the bioaerosols and limit our understanding of bioaerosol emissions. Besides, those methods can only provide snapshot data with low temporal resolution, which is difficult to capture the true nature and magnitude of bioaerosol emissions from WWTPs (Mainelis, 2020; Šantl-Temkiv et al., 2020). Additionally, poor repeatability (affected by the culture environment, e.g., temperature, relative humidity), selectivity to certain species, and labor intensiveness are also the disadvantages of cultivation method (Griffiths and DeCosemo, 1994; Heidelberg et al., 1997; Chi and Li, 2007; Korzeniewska, 2011).

To summarize, those existing methods are limited in providing the information on the size, fate and behavior of bioaerosol particles. Therefore, there is a need for improving the understanding of the temporal variation of the nature, magnitude and size distribution regarding to different processes in WWTPs, which can greatly contribute to the risk analysis modelling thus improving public health applications and management.

## Real time detection and characterization of bioaerosol emissions

In recent years, significant technological advancements have been made to develop rapid detection and characterization methods for bioaerosols, such as flow cytometry in conjunction with fluorescent technique (FCM/FL), laser induced breakdown spectroscopy (LIBS), laser/light-induced fluorescence (LIF), biochemistry and molecular biology analysis, aerosol mass spectrometry (MS) focusing on physical, chemical, and biological characterization of bioaerosols (Chen and Li, 2007; Steele et al., 2008; Ghosh et al., 2015; Druckenmüller et al., 2017; Fennelly et al., 2017; Nasir et al., 2018; Zhang et al., 2019; Negron et al., 2020; Šantl-Temkiv et al., 2020; Bhardwaj et al., 2021). Typically, each method offers different information on the complex mixture of atmospheric bioaerosls. For instance, LIBS and MS provides elemental composition of the particles in comparison to direct analysis of biochemical composition by different biochemistry and molecular biology analysis. Whilst LIBS and MS can rapidly record the elemental composition of single particles, detection and discrimination of biological materials is limited. Similarly, biochemical analysis can offer high sensitivity and selectivity but these are labor intensive and provide data with low temporal resolution. However, among these techniques, fluorescence spectroscopy has shown great potential to detect and broadly classify bioaerosols non-destructively in real time. Instruments based on LIF and/or elastic scattering have shown their capability and utility to detect and characterize bioaerosols in real-time in a range of ambient environments and sources (Nasir et al., 2019).

Briefly, the LIF based instruments interrogates the characteristic intrinsic fluorescence emission of particles and record the size, shape, and fluorescence spectra of single particles with high time resolution. These have been deployed into both laboratory and field studies. For example, Ultraviolet Aerodynamic Particle Sizer (UV-APS), Wideband Integrated Bioaerosol Sensor (WIBS) series (Healy et al., 2014; Oconnor et al., 2014; Perring et al., 2015; Crawford et al., 2016), Rapid-E (Šikoparija, 2020), Swisens Poleno (Sauvageat et al., 2020) and Spectral Intensity Bioaerosol Sensor (SIBS), have been employed in the bioaerosol monitoring in different environments (Nasir et al., 2018, 2019).

The LIF based devices present spectral resolution in the form of excitation-emission matrix (EEM; Pöhlker et al., 2012). There are three-excitation wavelength bands, which are the most commonly, used for distinguishing bioaerosol particles (Huffman and Santarpia, 2017). The excitation at approximately 255**–**285 nm band has been utilized to distinguish certain amino acid residues (Pöhlker et al., 2012). The excitation at approximately 340–370 nm band has been shown to promote fluorescence from the ubiquitous biological coenzyme biofluorophore NADH (Kaye and Hirst, 2011). Some LIF devices also use 450 nm to excite riboflavin and a variety of flavoproteins in bioaerosol particles (Pan, 2015).

The use of dual-wavelength excitation (e.g., 285 or 280 nm, and 370 nm) and the measurement of fluorescence in broad emission detection bands along with size and shape of single particles in real time is most prevailing approach. However, it is difficult to discriminate between different types of bioaerosols based on broad fluorescence emission detection bands (Pöhlker et al., 2012). The detection systems comprised of dual wavelength excitation and generating highly resolved spectral information of single particles in real time have been developed to overcome this challenge. For instance, SIBS is able to provide highly resolved spectral information (measuring fluorescence emission spectra in 16 wavelength bands) as compared to broad emission bands in the WIBS and other LIF based devices (Nasir et al., 2018, 2019; Könemann et al., 2019). Table 2 presents a comparison of parameters for different LIF based bioaerosol detection systems.

**Table 2:** Comparison of parameters for different LIF based bioaerosol detection systems.

	SIBS	WIBS-5	WIBS-4A	Rapid-E
Measured parameters	1. Particle size 2. Asphericity 3. Fluorescence spectra	1. Particle size 2. Asphericity 3. Integrated fluorescence in three channels	1. Particle size 2. Asphericity 3. Integrated fluorescence in three channels	1. Particle size 2. Particle shape (scattering images) 3.Fluorescence spectra
Particle size range	⁓ 0.5–30 μm	⁓ 0.5–30 μm	⁓ 0.5–31 μm	0.5–100 μm
Fluorescence excitation (*λ*_ex_)	285 and 370 nm	280 and 370 nm	280 and 370 nm	320 nm
Fluorescence emission bands (*λ*_em_)	*λ*_mean_ = 302–721 nm (16-channels)	310–400 nm and 420–650 nm	310–400 and 420–650 nm	350–800 nm (32 channels)

The application of these in field measurements in WWTPs are rare. Li et al. (2016) and Hsiao et al. (2020) both employed UV-APS for the detection of fluorescent particles and reported that the peak concentration of fluorescent particles was centered at 3–4 μm, which suggests the contribution from bacterial aggregates or fungal spores. Whilst, Tian et al. (2020) found the predominant size range is 0.5–1 μm by utilizing a SIBS. This is probably due to the differences in the excitation and emission bands. The UV-APS has 355 nm excitation wavelength and records emission at 420–575 nm to detect fluorescent particles in comparison to dual wavelength excitation and multi-channel emission measurements of SIBS. The highly resolved fluorescence spectra provided by SIBS may have the potential to elucidate the contribution of bioaerosols to total particles, the impact of various processes specific activities and the biological materials associated with airborne particles at WWTPs. Such investigations of bioaerosol emissions characteristics from WWTPs are of great value to better understand the size distribution and composition of emission profiles. This is critical for advancing and improving modelling methods to simulate dispersion of bioaerosols from WWTPs and the resultant health and environmental impacts.

To summarize, the high temporal resolution of size and number concentration data could greatly enhance the understanding of fate and behavior of bioaerosols, and likely deposition in the human respiratory tract. In combination with dose response studies, it has potential to better unravel the health effects of bioaerosols. Concurrently, high time resolution data could improve the understanding of transient emission dynamics, diurnal and annual cyclical variability of bioaerosol emissions, thus informing management strategies for bioaerosol emissions.

## Challenges for real time detection and characterization of bioaerosols

Whilst LIF based instruments have the potential to instantaneously detect airborne biological materials and provide an overall contribution of bioaerosols to total particles, there are still challenges with regards to confidence in their ability to discriminate biological and non-biological particles and categories biological particles. To begin with, fluorescent particles are not equivalent to viable bioaerosols, though live unculturable microorganisms can be detected, however the dead but morphologically intact microorganisms can also be quantified by fluorescence-based methods (Burdsall et al., 2021). Similarly, fluorescence from interfering non-biological compounds also make the discrimination of particles challenging. Hence, the development of more effective fluorescence threshold strategies will enable to filter the interfering particles and maximize the proxy for bioaerosols. There is also a need for developing standard reference materials for fluorescence measurements and intercomparison of LIF based measurements.

Following on, to enhance selectivity and discrimination of bioaerosol emissions, the highly resolved fluorescence intensity measurements (such as with SIBS) can help to discriminating between different biological particle types depending on their biofluorophore signatures due to significantly resolved spectral resolution. However, the key limitation is the information provided by resolved emission spectra requires meaningful interpretation (Könemann et al., 2019; Nasir et al., 2019). The assignment of fluorescence to specific biological fluorophores within atmospheric particles is challenging due to the complexity of the molecular environment (Pan, 2015) and the overlap of mixed signals from different fluorophores (e.g., mineral dust, polycyclic aromatic hydrocarbons, humic-like interferences; Pöhlker et al., 2012). Advanced data analysis ecosystems need to be developed to improve the discrimination and processing speed of analysis along with lab studies to improve certainty and validation of assigning spectral signatures to atmospherically relevant biological fluorophores.

## Conclusion and future directions

Overall, bioaerosol emissions from WWTPs presents a multicomponent/heterogeneity in nature, and highly variable in magnitude and particle size distribution with reference to different wastewater treatment unit and processes, which is of increasing health concern of nearby residential and occupational settings along with the urbanization process and population growth.

The advancements in real-time detection methods have shown the potential to overcome the methodological constraints enabling to detect and characterize the temporal variability of bioaerosols. However, long-term field investigations will help to better understand the dynamics of bioaerosol emissions from WWTPs and efficiently resolve public health issues relating to wastewater and bioaerosols. Depending on the treatment methods and type of wastewater, the categories and level of bioaerosol emissions could vary greatly. Hence, it is vital to conduct laboratory studies with biofluorophores, biological and non-biological particles in conjunction with other complementary methods (such as fluorimetry, gas chromatography, molecular methods) as well as *in situ* measurement to develop and validate library/network, database, and selective assignment of spectral responses to bioaerosol classes, for better discriminating bioaerosol particles and identifying the hazards with regards to various emission sources. In addition, studies on inter-technique comparability of fluorescence-based detection with advanced molecular methods will help to evaluate their reliability and utility to inform bioaerosol risk management at WWTPs. Simultaneously significant progress is required in the development of data analytics for optimizing fluorescence thresholds and discrimination between different biological particles.

Moreover, long-term *in situ* measurements will help to probe the temporal variation in the concentration and size distribution of bioaerosols to inform regulations concerning with occupational exposure and take measures to effectively mitigate bioaerosol emissions (e.g., avoid exposing to the time period with higher concentrations of emissions). A holistic system approach, involving multiple disciplines such as environmental science, engineering, microbiology, aerosol science, toxicology and epidemiology will pave the way to advance the knowledge on the factors and mechanisms influencing bioaerosol emissions and resultant health impacts. Process-based quantitative microbial risk assessment (QMRA) and dose response studies will also advance the evidence base on the exposure risk of bioaerosols released from WWTPs. Additionally, studies focusing on investigating the interaction of bioaerosols with abiotic components including other air pollutants will help to better understand the transformation, and the governing influences on viability, toxicity, and infectivity during airborne transport. This will inform the studies on exposure assessment and mechanism of toxicity enhancing the evidence base to better understand the dose–response relationships.

Advancement in detection and characterization of bioaerosols is critical in gaining insights into physicochemical and biological characteristics of emissions from WWTPs and elucidating their impacts in the context of public health (allergenicity, toxicity, and infectivity). This new knowledge will underpin the development of proportionate risk assessment and management policies and strategies to protect public health while ensuring the development of wastewater treatment infrastructure.

## Data availability statement

No new data were generated or analysed during this study.

## Author contributions

JT: investigation, formal analysis, methodology, visualization, and writing—original draft. CY: investigation, methodology, and writing—review and editing. SA: review and editing. FH: methodology and writing—review and editing. ST: funding acquisition and writing—review and editing. FC: funding acquisition, supervision, and writing—review and editing. ZN: conceptualization, supervision, methodology, administration, resources, and writing—review and editing. All authors contributed to the article and approved the submitted version.

## Funding

This paper received the financial support from the UK Research and Innovation (UKRI) Natural Environment Research Council (NERC) through the Environmental Microbiology and Human Health Programme (Grant references NE/M01163/1 and NE/M010961/1) and the UKRI Strategic Priorities Fund (SPF) Clean Air Programme (Grant reference NE/V002171/1). CY’s academic visit at Cranfield University was supported by the National Natural Science Foundation of China (Grant reference 51608497).

## Conflict of interest

The authors declare that the research was conducted in the absence of any commercial or financial relationships that could be construed as a potential conflict of interest.

## Publisher’s note

All claims expressed in this article are solely those of the authors and do not necessarily represent those of their affiliated organizations, or those of the publisher, the editors and the reviewers. Any product that may be evaluated in this article, or claim that may be made by its manufacturer, is not guaranteed or endorsed by the publisher.
